# Rare case of solid pseudopapillary neoplasm of the pancreas with liver and splenic metastasis in a 19-year-old girl

**DOI:** 10.1016/j.ijscr.2024.109867

**Published:** 2024-06-06

**Authors:** Chi-Chi Chen, Ting-Yuan Feng, Hsiang-Chun Jan, Shaw-Jiun Chou, Tzu-Hung Chen, Sheng-Chun Wang

**Affiliations:** Division of General Surgery, Department of Surgery, Cardinal Tien Hospital, New Taipei, Taiwan

**Keywords:** Pseudopapillary, Neoplasms, Frantz tumor, Pancreas, Case report, Pancreatectomy

## Abstract

**Introduction:**

Solid pseudopapillary neoplasms (SPNs) of the pancreas are rare neoplasms, accounting for only 1 %–2 % of all pancreatic tumors, and predominantly affect female patients.

**Case presentation:**

The present case report details a patient presenting to the emergency department with abdominal pain for 3 days who ultimately received a diagnosis of SPNs in the pancreatic body and tail. A contrast-enhanced computed tomography (CT) scan revealed a sizable mass arising from the pancreas, featuring an enhancing cystic component with involvement of the liver and spleen. The patient underwent subsequent exploratory laparotomy, a distal pancreatectomy, splenectomy, and partial hepatectomy. SPN diagnosis was confirmed by histopathology and immunohistochemistry with negative resection margins.

**Clinical discussion:**

Approximately 70 % of SPN cases are asymptomatic and are incidentally discovered. Despite advances in diagnostic modalities, preoperative diagnosis of SPNs remains a clinical challenge. Surgical management with negative resection margins remains the primary treatment approach. The recurrence rate after surgical resection has been reported to be 3 %–9 %. The prognosis for SPNs limited to the pancreas is generally favorable, with a cure rate exceeding 95 % after complete surgical resection.

**Conclusion:**

An SPN of the pancreas is a rare tumor observed in young female patients. Although it is classified as a malignant tumor, SPN has low malignant potential. Aggressive surgical resection, however, has proven effective in curing SPN for the majority of patients.

## Highlights

• A solid pseudopapillary neoplasm of the pancreas is a rare tumor observed in young female patients.
• Although it was categorized as low malignant tumors, SPNs have been associated with reports of metastasis.
• Surgical management with negative resection margins remains the primary treatment approach.

## 1 Introduction

*Epidemiology:* Solid pseudopapillary neoplasms (SPNs) of the pancreas, also known as Frantz tumors after Virginia K. Frantz, who first described these neoplasms in the 1950s, received its official nomenclature from the World Health Organization in 1996 [1,2]. SPNs account for a small percentage of pancreatic tumors (0.17 % to 2.7 %) [3] and typically affect young adult female patients (female to male ratio of 11:1) in their second or third decade of life.

*Clinical presentation:* Frequently discovered incidentally, these tumors manifest as slow-growing abdominal masses with nonspecific symptoms, such as abdominal pain. The tumor's predominant location is in the body or tail of the pancreas (60 % of cases); however, it may also be located in the pancreatic head [1,3,6]. Approximately 70 % of cases are asymptomatic, and SPNs are often detected incidentally.

*Diagnostic challenges:* Despite advances in diagnostic modalities, achieving a preoperative diagnosis of SPNs remains a clinical challenge. Elevated levels of tumor markers such as alpha-fetoprotein, carcinoembryonic antigen, CA199, CA125, and CA242 may be observed; however, these markers lack specificity for SPNs [6].

*Treatment options:* Surgical management in which free surgical resection margins are achieved remains the primary treatment approach for SPNs. However, in cases in which lesions are deemed unresectable, surgical debulking might be justified [3,8]. Although SPNs are known for their radiosensitivity, chemotherapy has also led to promising results [5]. However, the effectiveness of radiotherapy and chemotherapy in such cases remains a subject of investigation, and such treatments are only considered alternatives in cases in which surgical intervention is contraindicated [5,24].

*Prognosis:* The prognosis for SPNs confined to the pancreas is generally excellent, with a cure rate exceeding 95 % after complete surgical resection [5]. Furthermore, local invasion and metastasis are not considered contraindications for surgical resection. The present report details the case of a 19-year-old girl given a diagnosis of SPN and a review of the literature on this rare type of tumor.

## 2 Case

*History and Physical examination:* A 19-year-old girl with no relevant medical or surgical history presented to our emergency department due to vomiting and acute abdominal pain localized in the left upper quadrant region for the past week. The patient reported no history of jaundice, weight loss, night sweats, pruritis, urinary discoloration, constipation, or diarrhea. Additionally, she had no pertinent surgical history, and her family history did not reveal any instances of pancreatic disease. Upon physical examination, tenderness was noted in the left hypochondrial region without the presence of a palpable mass.

*Diagnostic process:* 1. Laboratory investigations did not indicate any evidence of pancreatic insufficiency, abnormal liver function, cholestasis, or elevated pancreatic enzymes. Furthermore, all tumor markers, including alpha-fetoprotein (AFP), carbohydrate antigen (CA 19-9), and carcinoembryonic antigen (CEA), were within normal ranges. WBC 16.23 x 10^9^/L, Hb 13.2 g/dl, platelet 167 x 10^9^/L, band 0, BUN 15 mg/dl, Creatinine 0.63 mg/dl, Na 133 mEq/L, K 3.61 mEq/L, Lipase 18 U/L, GPT 16 U/L, CRP 0.327 mg/dl, CEA 0.66 ng/ml, CA-199 5.44 U/ml, CA-125 19.9 U/ml, AFP 1.4 ng/ml.

2. A computed tomography (CT) scan revealed a large heterogeneous mass with both solid and cystic components, measuring approximately 6.7 × 6.2 × 3.7 cm^3^. The mass was located at the pancreatic tail, with invasion of the tumor to the splenic and portal veins. Additionally, the scan revealed hepatosplenomegaly accompanied by multiple hypoenhanced masses in both the liver and spleen (Fig. 1-1, Fig. 1-2).

**Fig. 1-1:**
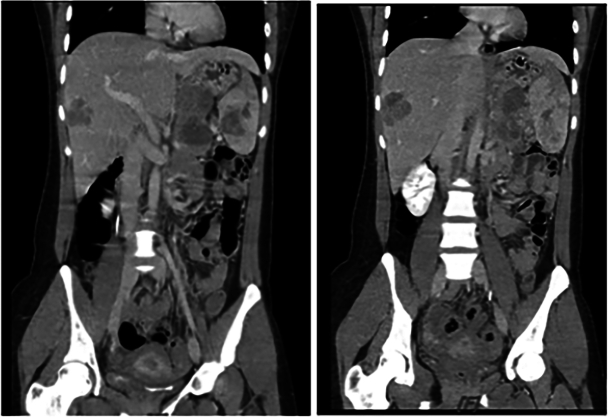
Pancreatic tail tumor with splenic vein and portal vein thrombosis, multiple liver and spleen metastasis, and ascites (abdomen computed tomography: coronal view).

**Fig. 1-2:**
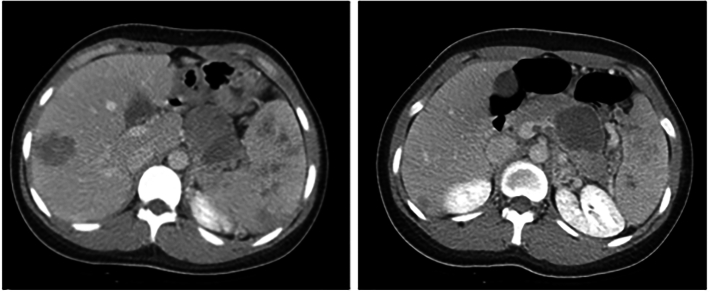
Pancreatic tail tumor with splenic vein and portal vein thrombosis, multiple liver and spleen metastasis, and ascites (abdomen computed tomography: axial view).

**Fig. 2:**
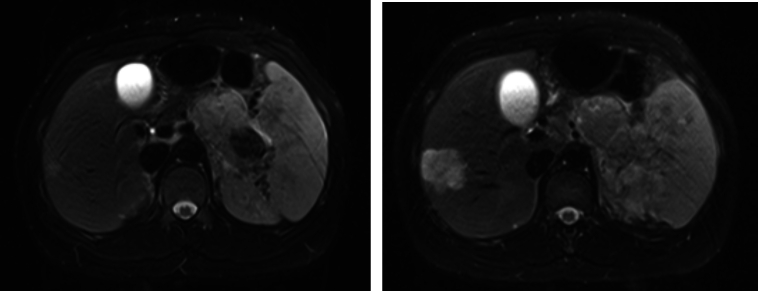
Axial view from a T2-weighted MRI revealing a suspected tumor in the pancreatic body and tail, with liver and splenic metastases.

3. To confirm the diagnosis, the patient underwent a magnetic resonance imaging (MRI) scan, which corroborated the previously identified findings. The MRI scan indicated a suspected tumor located in the pancreatic body and tail, with accompanying metastases detected in the liver and spleen (Fig. 2).

4. To confirm the presence of distant metastasis, a whole-body bone scan, brain MRI, and chest CT were arranged. No evidence of metastasis to the bones, brain, or lungs was observed.

5. Before the patient underwent elective surgery, an ultrasound-guided liver biopsy was performed, and the histopathological examination revealed a metastatic liver tumor exhibiting a papillary configuration. Immunohistochemical analysis revealed that the tumor cells were positive for CD56, synaptophysin, and beta-catenin but exhibited no reactivity for E-cadherin.

6. Considering the patient's clinical history of pancreatic tumor, the morphological and immunohistochemical features observed in the liver biopsy were considered indicative and compatible with a diagnosis of metastatic SPN of the pancreas.

*Treatment:* Before the surgical decision was made, the case was discussed in MDT. After discussing with family members and patient, they decided to undergo elective surgery with further treatment plan (whether the need of TAE or chemotherapy) according to pathological result. Given the favorable prospect of resecting the tumor, a decision was made to proceed with the excision of the mass. Approximately 3 weeks later, the patient underwent elective surgery to remove the tumor.

**Fig. 3:**
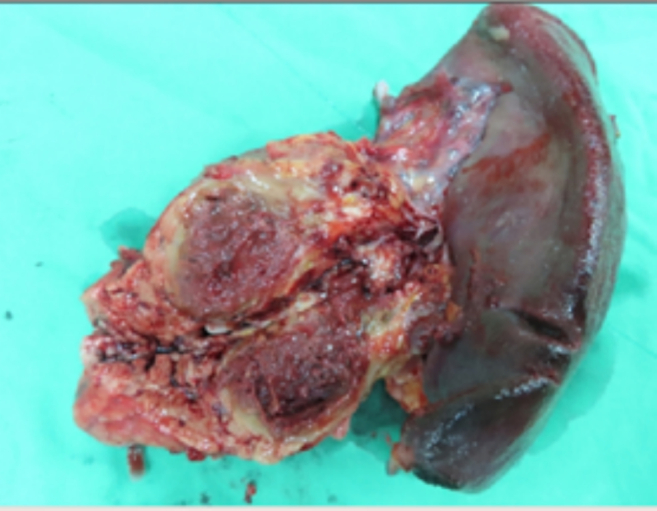
Excised specimen; large mass detected in the pancreatic tail, with splenic involvement.

During the exploratory laparotomy, a large mass was discovered in the pancreatic tail with evident splenic involvement and portal vein thrombosis. To address this, a distal pancreatectomy and splenectomy were performed (Fig. 3). Additionally, palpable liver masses were resected during the same operation. The surgical approach also involved segmentectomy of the liver (Segment 2, Segment 3) and partial hepatectomy (Segment 4a, 5, 6). A total of three liver tumors, with sizes up to 2.3 × 1.5 × 1.2 cm^3^ in the right lobe of the liver, were successfully resected.

Histopathological examination revealed an ill-defined, solid, gray, and focally hemorrhagic tumor characterized by polygonal cells exhibiting pseudopapillary formations. This confirmed the diagnosis of an SPN of the pancreas (Fig. 4a, Fig. 4b). Additionally, three tumor nodules were identified in segments 2 and 3 of the liver that measured up to 0.6 × 0.6 × 0.5 cm^3^, 1.8 × 1.5 × 1 cm^3^, and 1.8 × 1.5 × 1 cm^3^. Immunostaining supported the diagnosis, indicating reactivity in CD10 (Fig. 5c) and alpha-1-antichymotrypsin (Fig. 5d). Hyalinized acellular stroma (Fig. 5a) and negative with cyokeratin (CK) stain (Fig. 5b).

**Fig. 4a:**
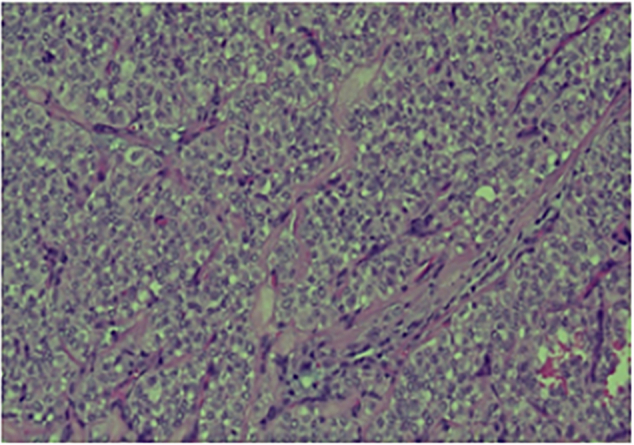
Papillary configuration.

**Fig. 4b:**
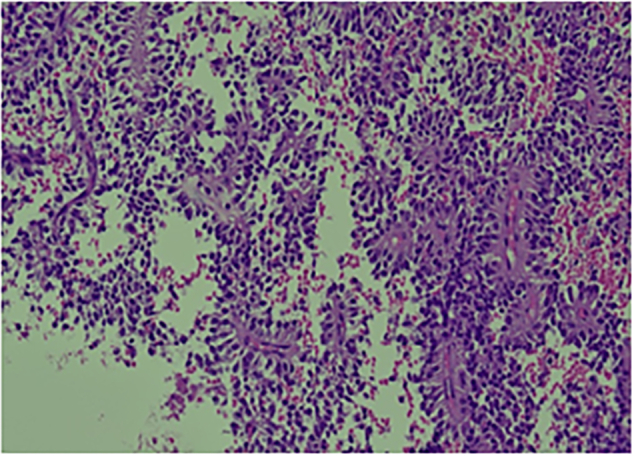
Solid pseudopapillary neoplasm of the pancreas.

**Fig. 5a:**
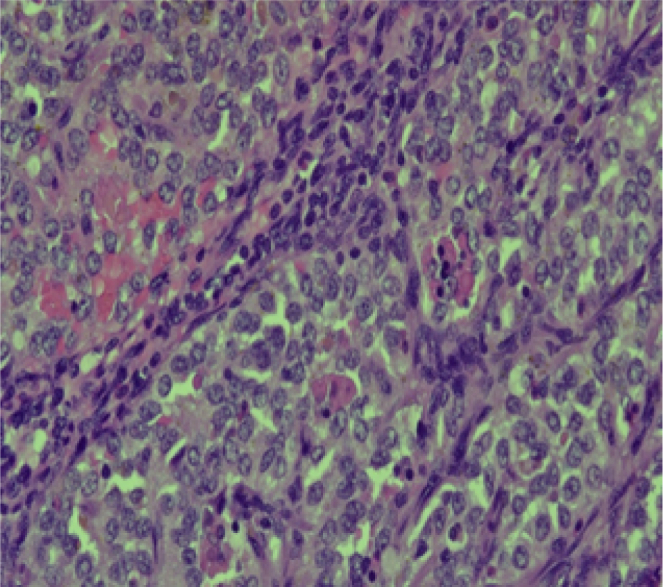
Hyaline globulin (+).

**Fig. 5b:**
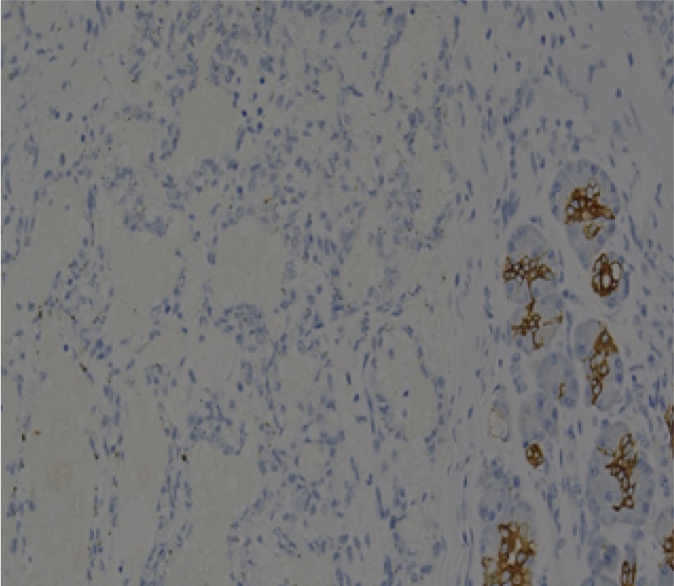
CK (−).

**Fig. 5c:**
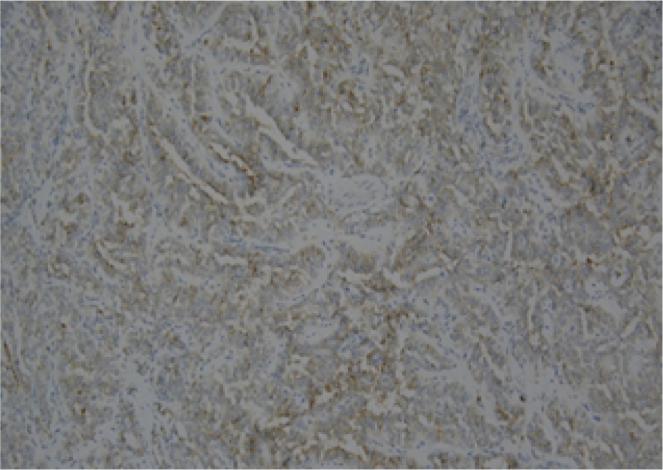
CD 10 (+).

**Fig. 5d:**
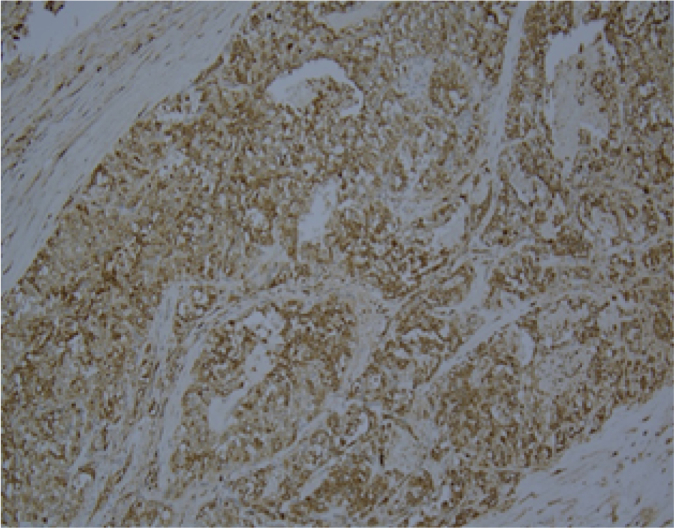
Alpha 1 antitrypsin (+).

Histopathological examination indicated reactive hyperplasia of the lymph node with no evidence of lymphovascular or perineural invasion. The tumors were determined to be stage IV, according to the Pathological TNM classification system, with the following details: T:4, N:0, M:1. Despite the advanced stage, the patient experienced a smooth and uneventful postoperative course, leading to eventual discharge.

*Follow up:* After surgical intervention, the patient received adjuvant chemotherapy with FOLFIRINOX for 4 cycles (3 weeks interval). (FOLFIRINOX: Folinic acid, 5-FU, Irinotecan, Oxaliplatin). FOLFIRINOX regimen was emerged in 2010 as a new treatment for patients with metastatic pancreatic cancer. However, FOLFIRINOX is a potentially highly toxic combination of drugs with serious side effects, and only patients with good performance status are candidates for the regimen. During follow up, recurrence of liver metastasis was noted 4 months after surgery. As the patient denied to receive further chemotherapy, the patient lost followed up.

## 3 Discussion

*Epidemiology:* An SPN is a rare tumor and accounts for a small percentage of all pancreatic tumors, with reported percentages ranging from 0.17 % to 2.7 % [3]. Although they rarely occur, an increasing number of cases of SPNs have been detected because of advancements in imaging modalities. A review conducted by Law et al. [11] identified 2744 cases of SPN, with more than 87 % of the cases having been reported post-2000. The review indicated that the number of reported cases was higher by seven-fold in 2000–2012 than in 1961–1999. In addition, men accounted for only 12.2 % of the cases. The increase in the number of reported cases can be attributed to increased awareness and improved diagnosing capabilities rather than an actual increase in incidence. SPNs typically affect young adult female patients in their second or third decade of life, with the female to male ratio for SPNs being 11:1 [[4], [5], [6]]. The higher prevalence among female patients was hypothesized to be associated with the close proximity of primordial pancreatic cells to the ovarian ridge during embryonic development [12]. Although SPNs are most commonly diagnosed in young female patients, cases have also been reported in children [13], older adults, and men [14]. The most common location of SPNs is the tail or body of the pancreas.

*Pathophysiology:* Despite various proposed hypotheses, the pathophysiology and cellular origin of SPNs continue to be subjects of debate [5,9,10]. SPN cells express beta-catenin, E-cadherin, vimentin, alpha-1 antitrypsin and alpha-1 chymotrypsin, CD10, and CD56. Conversely, they test negative for pancreatic enzymes and chromogranin [17]. All SPNs exhibit mutations in the beta-catenin pathway that involve the CTNNB gene [18]. These mutations can be identified in fine needle aspirates of the tumors [19]. Despite being characterized as slow-growing tumors with a low Ki-67 index, the doubling time of SPNs can vary considerably, as indicated in several case reports, ranging from 240 to 765 days [23,24]. This wide range suggests that although these tumors are generally slow-growing, their growth rate significantly varies [23].

*Diagnostic modalities:* Laboratory findings associated with SPNs are generally inconspicuous, with no increase occurring in amylase or lipase levels and no elevation of tumor marker levels, a finding typically associated with pancreatic carcinomas. In a single-institution study by Beltrame et al. [20], which included 451 patients with pancreatic cystic tumors, only 18 (3.7 %) cases were histologically confirmed as SPN, and only 1 patient exhibited an elevated serum CA 19–9 level of 92 U/mL (normal range: 0 to 37 U/mL). Notably, beta-catenin expression was observed in all patients.

Preoperative diagnosis of SPNs remains a clinical challenge, despite advances in diagnostic modalities [8]. This is because of the potential overlap with a wide range of differential diagnoses, including benign cystic lesions, such as pseudocysts, hydatid cysts, cystadenoma, lymphangioma, and hemangioma, and malignant lesions, such as cystadenocarcinoma and intraductal papillary mucinous neoplasms [3,5]. In the pediatric age group, pancreatic tumors of secondary origin, including neuroblastoma, leukemia, lymphoma, and lymphoproliferative disorders, are more common [5].

In cases of SPN, elevated levels of tumor markers such as alpha-fetoprotein, carcinoembryonic antigen, CA199, CA125, and CA242 may be observed; however, these markers lack specificity for SPNs [6]. Nevertheless, these tumor markers, along with pancreatic tumor markers, should be considered during diagnostic work-up because other malignant tumors are included in the differential diagnosis of SPNs [8].

In terms of imaging, an abdominal CT scan with intravenous contrast was identified as the optimal imaging technique for assessing SPNs because it provides information regarding the origin, size, and configuration of the tumor and information related to local invasion and the presence of metastasis [5]. Because SPNs have a mix of solid and cystic components, CT scans typically depict areas with both enhancing and nonenhancing lesions surrounded by a capsule and exhibiting intratumoral calcifications [5]. Furthermore, hemorrhage may result in cases of SPN due to the growth of the tumor and subsequent internal degeneration [25]. The presence of an encapsulated mass consisting of both cystic and solid components and intratumoral hemorrhage serve as factors distinguishing SPNs from other malignant differentials [5,9,25]. Because they enable identification of these pathognomonic features of SPNs, CT scans are considered adequate for establishing a preoperative diagnosis [26].

MRI is considered a secondary imaging modality for SPNs because it can provide insights into hemorrhaging and the necrosis of tumor tissue [26]. In the context of SPNs, MRI results commonly reveal a vascular, encapsulated mass composed of mixed cystic and solid components characterized by a high signal intensity on T1 and low signal intensity on T2, which represent hemorrhagic areas [25]. However, Dan et al*.* indicated that MRI was not required in their reported cases of SPNs located in the tail of the pancreas; CT scans were effectively able to reveal the pathognomonic features of SPN [26].

Endoscopic ultrasound (EUS) and endoscopic retrograde cholangiopancreatography (ERCP) play a role in further clarifying the diagnosis of SPNs [27]. Weaver et al. [28] reported that the addition of endoscopic ultrasound-guided fine needle aspiration (EUS-FNA) to a preoperative work-up of SPN led to an increase in diagnostic yield of up to 82.4 %. However, some practitioners may hesitate to perform fine needle aspiration because of the potential risk of peritoneal dissemination and associated complications. Although 70 % of all cases of SPNs are symptomatic, incidental discovery occurs in 30 % of cases [21]. As the tumor enlarges and exerts pressure on adjacent organs, most patients complain of abdominal pain that is often accompanied by an increase in abdominal girth.

Surgical management in which free surgical resection margins are achieved remains the primary treatment approach for SPNs. This holds true even in cases with metastasis and vascular invasion, where surgical excision is recommended whenever feasible [8]. Radical lymphadenectomy is not indicated in these cases [5]. The reported recurrence rate following surgical resection ranges from 3 % to 9 % [9], indicating a need for prompt follow-up due to the potential risk of recurrence or emergence of metastatic lesions [24]. In our case, recurrent liver masses were noted after 4 months of surgical resection and were stationary. This indicates that even in cases of recurrence or metastasis, surgery remains the treatment of choice. However, in cases in which lesions are deemed unresectable, surgical debulking might be justified [3,8].

Two forms of resection may be employed, depending on the tumor's location. If the tumor is situated in the body or tail of the pancreas, distal pancreatectomy with or without splenic preservation is the recommended surgical approach [5]. Conversely, when the tumor is located in the head of the pancreas, pancreaticoduodenectomy is the preferred method [5]. During surgical resection, meticulous care must be given to prevent rupture or spillage of the tumor content. Such events could lead to the seeding of tumor cells into the peritoneum [7]. Given the encapsulation and generally low malignant potential of SPNs, surgical management should be performed as conservatively as possible [5].

The presence of metastases is not considered a contraindication to surgical removal in the management of SPNs [29]. Surgical resection of metastases, either concurrently with or following the resection of the primary tumor, is compatible with achieving long-term disease-free survival. However, certain features, such as perineural invasion, angioinvasion, and invasion of neighboring structures and tissues, along with characteristics such as a large size, cellular or nuclear atypia, and a high mitotic rate have been associated with increased malignant potential and a higher rate of recurrence of SPNs [22]. The major sites of metastases in SPNs are the liver and peritoneal cavity [9,25]. Although SPNs are known for their radiosensitivity, chemotherapy has also led to promising results [5]. However, the effectiveness of radiotherapy and chemotherapy in such cases remains a subject of investigation, and such treatments are only considered alternatives in cases in which surgical intervention is contraindicated [5,24].

Because most of tumors contain estrogen and progesterone receptors, there may be a role for systemic tamoxifen when surgical resection is not an option. As for clinical practice, there are report of successful Tamoxifen treatment with accounts of a stable disease maintained for 12 years in a patient with unresectable local disease, as well as evidence of antiestrogen drug being effective in patient with liver metastasis. Despite, the evidence of using endocrine therapy in real clinical practice is still very limited. Currently, there are no clinical studies on this issue, and the data is limited to several case reports [15,16]. There was also treatment using cryoablation plus interventional embolization, which could be a promising alternative therapy for pancreatic SPT liver metastasis although there was limited case studies and datas [33].

*Prognosis:* The prognosis for SPNs confined to the pancreas is generally excellent, with a cure rate exceeding 95 % after complete surgical resection [5]. Furthermore, local invasion and metastasis are not considered contraindications for surgical resection.

An essential aspect of managing SPNs is predicting which patients may experience recurrence and determining the appropriate postsurgical follow-up. Serrano et al. [30] revealed that recurrence often occurs 5 to 7 years after complete surgical resection, highlighting the need for a clinical follow-up exceeding 5 years, with routine imaging being particularly crucial for high-risk patients exhibiting features such as lymphatic and blood vessel invasion, metastasis, and potential invasion of the tumor capsule [31]. A meta-analysis [32] reported a postresection recurrence rate of 2 %, identifying male patients and those with positive lymph nodes, R1 margins, and lymphovascular invasion as being more prone to recurrence.

## 4 Conclusion

SPN of the pancreas is a rare tumor observed in young female patients, suggestive of possible role of hormonal factors. Preoperative diagnosis of SPNs is by diagnostic imaging and biopsy. Aggressive surgical resection, has proven effective in curing SPN for the majority of patients. Surgical resection of metastases, either concurrently with or following the resection of the primary tumor, is compatible with achieving long-term disease-free survival. The prognosis for SPNs confined to the pancreas is generally excellent, with a cure rate exceeding 95 % after complete surgical resection. Therefore, from this case and literature review, we concluded that early diagnosis and treatment (surgical intervention) can improve the patient outcomes and better prognosis.

## Ethical approval

Include in IRB CTH-113-3-7-001

Ethical approval for this study (CTH-113-3-7-001) was provided by the Ethical Committee of Cardinal Tien Hospital, New Taipei City, Taiwan on 2 February 2024.

## Funding

Personal.

## Author contribution

Correspondence to: Sheng-Chun Wang

Writing the paper: Chi-Chi Chen

Guidance of all: Ting-Yuan Feng, Shaw-Jiun Chou, Hsiang-Chun Jan, Tzu-Hung Chen

## Guarantor

Chi-Chi Chen

## Methods section

The work has been reported in line with the SCARE criteria.

## Conflict of interest statement

The authors declare that they have no conflict of interest regarding the publication of this paper.
